# A fusion model based on tumor and peritumoral CT radiomics for differentiating bronchiolar adenoma from lung adenocarcinoma

**DOI:** 10.3389/fonc.2026.1798218

**Published:** 2026-04-15

**Authors:** Ziqian Zhao, Tengfei Ke, Zeyan Xu, Yong Zhou, Yanwen Dong, Yifan Liu, Jianping Wu, Wenyan Wei, Dan Han, Wen Zhao

**Affiliations:** 1 Department of Medical imaging, the First Affiliated Hospital of Kunming Medical University, Kunming, China; 2 Department of Medical imaging, Second people's Hospital of Qujing City, Qujing, China; 3 Department of Radiology, Yunnan Cancer Hospital, the Third Affiliated Hospital of Kunming Medical University, Kunming, China; 4 Department of first Thoracic Surgery, the First Affiliated Hospital of Kunming Medical University, Kunming, China

**Keywords:** bronchiolar adenoma, computed tomography, CT, lung adenocarcinoma, machine learning, radiomics

## Abstract

**Purpose:**

This study aimed to develop and validate a combined clinical and tumor–peritumoral CT radiomics model to differentiate bronchiolar adenoma (BA) from lung adenocarcinoma (LUAD), thereby improving preoperative diagnostic accuracy and guiding individualized treatment strategies.

**Methods:**

A total of 362 patients with pathologically confirmed BA or LUAD were retrospectively analyzed. Data from Medical Center 1 (n = 281) were divided into training and test sets (7:3 ratio), and data from Medical Center 2 (n = 81) served as an external validation set. Clinical characteristics, CT morphological features, and tumor–peritumoral radiomics features were extracted. Five machine learning algorithms were applied to construct and compare predictive models.

**Results:**

Lung lobe distribution, density, vacuolar sign, tumor-associated vessels, distance to pleura, and nodule diameters differed significantly between BA and LUAD. Among radiomics models, the tumor–peritumoral MLP model achieved the best performance (AUCs: 0.918, 0.912, 0.888). The clinical–radiomics fusion model outperformed single models, with AUCs of 0.935, 0.939, and 0.910 and accuracies of 0.862, 0.847, and 0.864 in the training, test, and validation sets, respectively.

**Conclusion:**

The proposed fusion model enables accurate, non-invasive differentiation between BA and LUAD, offering valuable support for personalized clinical decision-making.

## 1 Introduction

Bronchiolar adenoma (BA) is a rare benign pulmonary tumor originating from the mucosal epithelium of the bronchioles, with treatment options including regular follow-up observation or localized lobectomy (1). BA has characteristic structural features, including a bilayer of basal and luminal cells, which can be identified through immunohistochemical markers such as CK5/6 and P40 (2, 3). This structure is crucial for distinguishing BA from lung adenocarcinoma (LUAD), which typically presents with flat or acinar-like structures (4). However, this characteristic structure requires immunohistochemical observation (5), and BA is often misdiagnosed as LUAD during intraoperative frozen section analysis (6, 7). The misdiagnosis during frozen section can lead to an extension of the surgery, from the originally planned localized lobectomy to wedge resection or even pneumonectomy. Therefore, accurate preoperative differentiation of the bilayer cell structure of BA from the flat or acinar-like structures of LUAD is of utmost importance.

CT is an important preoperative examination for lung nodules, but differentiating BA from LUAD presents challenges due to the significant overlap in their CT imaging features (8, 9). One study focusing on the differentiation between BA and LUAD showed that the related CT features, including the maximum lesion diameter, average CT value, lung lobe distribution, lesion density, boundary, and morphology, had a sensitivity of 88.6%, but a specificity of only 66.7% (10), indicating that relying solely on morphological features cannot meet the clinical need for precise diagnosis. Radiomics uses high-throughput data analysis to reflect tumor heterogeneity, including both intratumoral features and changes in the peritumoral microenvironment (11, 12). Currently, there are limited studies on differentiating BA from LUAD. Liu et al. (13) suggested that intratumoral radiomics features can preliminarily differentiate between the two, but the sample size was small, and there was no external validation. Moreover, there are no studies based on peritumoral radiomics for this differentiation. As BA is a benign tumor with no peritumoral invasion, we believe that peritumoral radiomics may provide important information for distinguishing between BA and LUAD.

This study aims to establish a combined model incorporating clinical features, CT characteristics, and radiomics features from both the tumor and peritumoral regions using various ML algorithms. The goal is to provide a new non-invasive approach for preoperative differentiation of BA and LUAD, identify clinical and radiomics biomarkers of tumor and peritumoral microenvironment changes, and ultimately assist in formulating personalized treatment strategies for clinical practice.

## 2 Materials and methods

This study strictly adhered to the Declaration of Helsinki and was approved by the Ethics Committee (NO.2024-L-169). Informed consent was waived for the retrospective study.

### 2.1 Study sample

**Figure 1:**
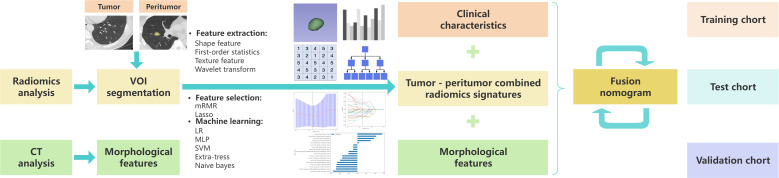
Overview of the study methodology.

**Figure 2:**
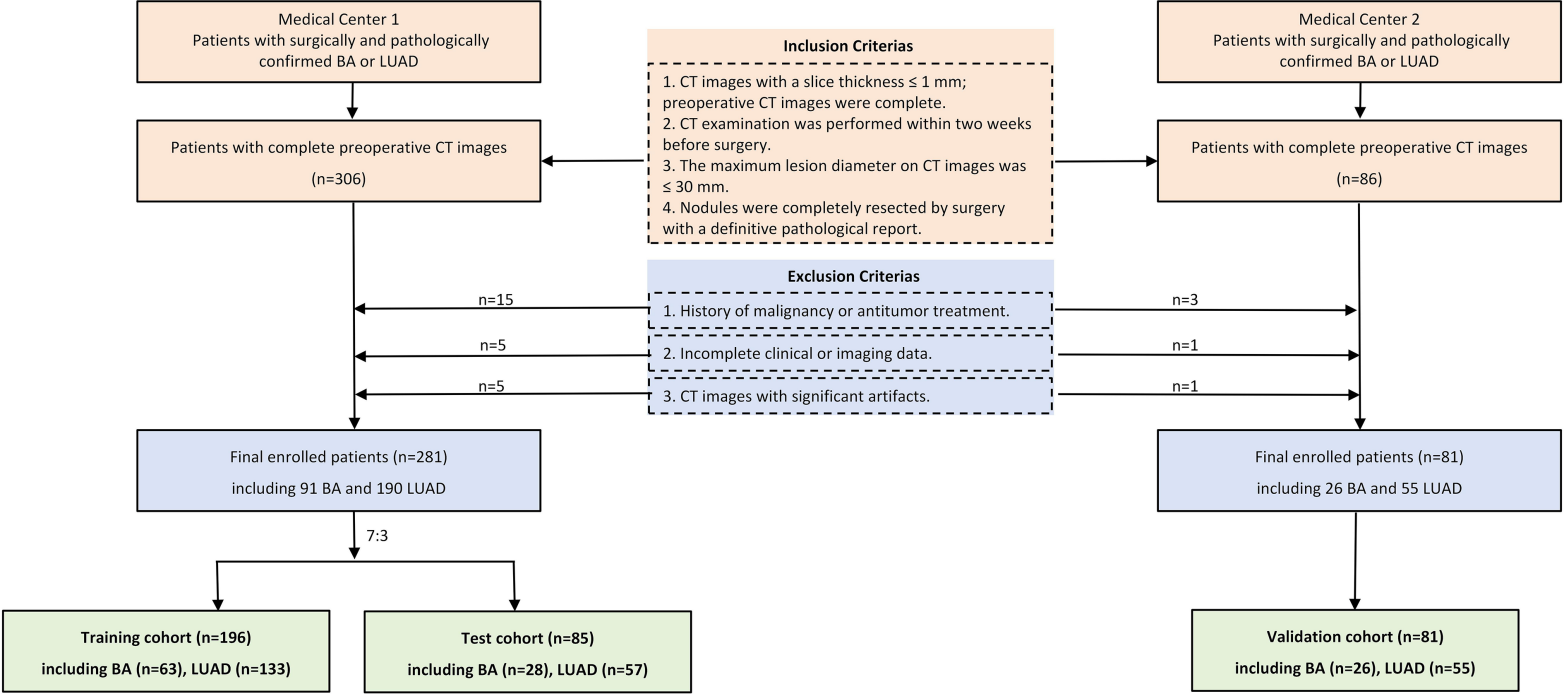
Flowchart shows participant inclusion and grouping.

Patients diagnosed with BA or LUAD through preoperative chest CT scans and confirmed by surgical pathology were retrospectively collected. Given the larger number of patients with LUAD, to balance the case numbers and enhance the scientific rigor of the study design, LUAD patients whose surgery dates were within approximately two days of the enrolled BA cases were selected at a ratio of about 1:2 (BA: LUAD). During case collection, if postoperative pathology indicated multiple nodules, only the largest nodule corresponding to the pathological findings and clearly discernible on CT images was included. Medical Center 1 included 281 patients (91 with BA, 190 with LUAD), consisting of 86 males and 195 females, with a mean age of 53.23 ± 12.67 years (ranging from 22 to 86 years). The cases were randomly divided into a training set (n = 196) and an internal test set (n = 85) in a 7:3 ratio. Medical Center 2 included 81 patients (26 with BA, 55 with LUAD), consisting of 25 males and 56 females, with a mean age of 53.85 ± 14.22 years (ranging from 24 to 76 years), and served as the external validation set. The overview of this study methodology is shown in **Figure 1**, and the flowchart of the participants grouping is shown in **Figure 2** including the inclusion criteria and exclusion criteria.

### 2.2 CT Examination methods

The scanning machines used in this study include the Siemens SOMATOM Definition Flash (Siemens Healthineers, Germany), Philips IQon Spectral CT (Koninklijke Philips N.V., Netherlands), Canon Aquilion ONE GENESIS (Canon Medical Systems, Tokyo, Japan), GE Revolution APEX CT (GE Healthcare, Milwaukee, WI, USA), and United Imaging uCT-760 (United Imaging Healthcare Co., Ltd., Shanghai, China). The patients were positioned in a supine position, with the scanning range from the apex of the lungs to the diaphragm angle. The scanning parameters were as follows: tube voltage of 100–120 kV, effective tube current of 200–250 mAs (automatic milliampere modulation), rotation time of 0.5–1.0 s, and a slice thickness and interval of 1.0 mm for thin-slice reconstruction. Images were analyzed using the lung window settings (window width 800 to 1500 HU, window level -600 to -800 HU).

### 2.3 Image analysis

**Figure 3:**
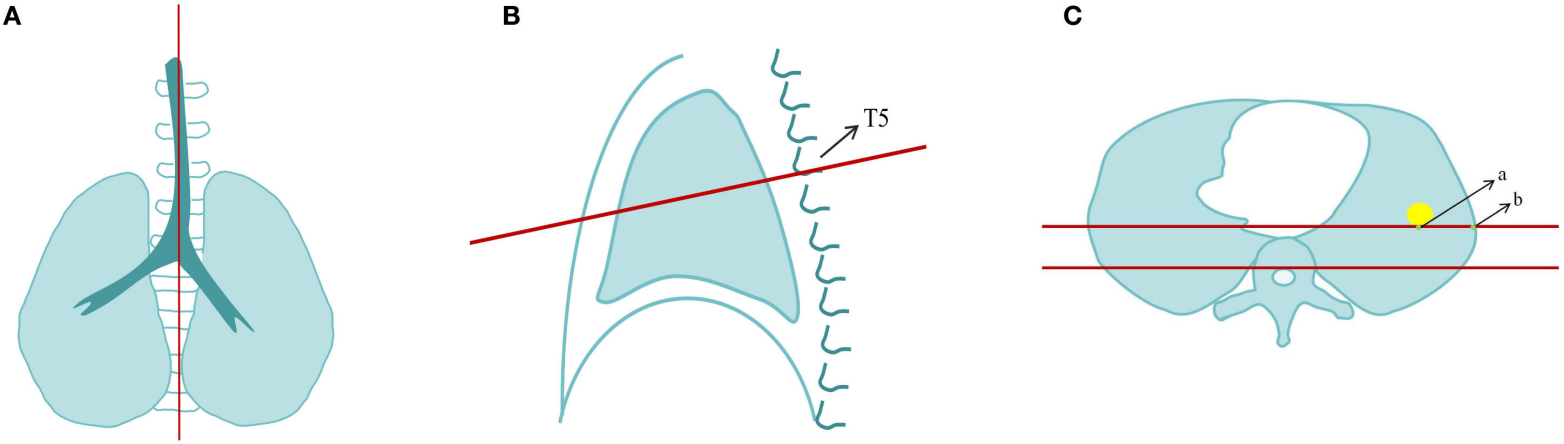
Standardized measurement process of distance from the pleura (DTP). Step 1: Adjust the reference line on the MPR as shown in **(A)**, where the long axis of the spine is used as the baseline to obtain the midsagittal plane image **(B)**. Step 2: In the sagittal plane **(B)**, use the lower edge of the T5 vertebra as the reference line to obtain the standardized axial plane image **(C)**. Step 3: In the axial plane **(C)**, select the maximum cross-sectional plane of the nodule. Draw a horizontal line at the posterior edge of the nodule (parallel to the posterior edge of the vertebra) and measure the distance between the nodule and the pleura along this horizontal line, which is the distance between points a and b (DTP). The measurement is repeated three times, and the average value is taken. If pleural retraction is present, the DTP is recorded as 0 mm.

Two radiologists with experience in chest imaging (3 years and 10 years, respectively) independently analyzed the images. In case of discrepancies, both radiologists discussed and reached a consensus. Using thin-slice multi-planar reconstruction (MPR) lung window images, they observed and recorded the CT characteristics and quantitative parameters of the nodules. The CT features included: density, location of lung lobe, bronchial sign, pleural retraction sign, vascular sign, vacuolar sign, cavity sign, spiculated sign, and lobulated sign. The quantitative parameters included: tumor-related vessels number (TVN), long and short diameters, and distance to pleura (DTP). To further minimize the influence of subjective factors on the results, the thin-section lung window CT images of all patients were imported into the Deepwise Pulmonary Nodule CT Image Assisted Diagnosis Software (version 2.4.0). The long-axis and short-axis diameters of the nodules, as well as their lobar distribution, as measured by the software, were recorded. Since previous studies showed significant subjectivity in DTP measurements, this study innovatively standardized the DTP measurement, as shown in **Figure 3**. Consistency was assessed for both this DTP measurement method and the conventional measurement method. It was found that the ICC for the conventional DTP measurement method was 0.69, whereas the ICC for our measurement method was 0.81. In the training set, univariate and multivariate logistic regression were used to identify independent factors for differentiating BA and LUAD, and a clinical-subjective model was developed based on these features.

### 2.4 Radiomics analysis

The thin−section CT lung window images of all included cases were imported into 3D Slicer software (version 5.2.2, http://www.slicer.org). Spatial resampling of the original images was performed using a linear interpolation algorithm to obtain standardized images with a voxel size of 1.0 mm × 1.0 mm × 1.0 mm. Chest radiologists manually delineated the region of interest (ROI) along the tumor boundary layer by layer on the lung window CT images. Furthermore, in accordance with the peritumoral analysis ranges frequently adopted in prior studies (11, 14, 15) on lung cancer and other malignancies, a ROI extending 3 mm automatically outward from the tumor boundary was defined as the peritumoral ROI. During manual delineation of the tumor ROI, vessels and bronchi were deliberately avoided. When automatically expanding the peritumoral ROI, if the resulting region included vessels, bronchi, chest wall, or pleura, these areas were manually removed. Using the “Radiomics” module, radiomics features for the tumor, peritumoral region, and tumor-peritumoral region (the combination of tumor and peritumoral features) were extracted. For feature extraction, two image types were defined: original images and images processed by wavelet transform (covering all combinations of high−pass and low−pass filters). In addition, to ensure consistency and comparability in feature extraction, a fixed bin width of 25 was set. For each low−frequency component obtained after wavelet transformation, denoising was performed using different scale thresholds, while the high−frequency components were processed by selecting the optimal coefficients based on local variance criteria. Thirty cases were randomly selected from the patient cohort using a computer random number generator. The same observer repeated the contouring one month after the initial delineation to assess intra-observer agreement. Concurrently, another radiologist with 10 years of experience in thoracic imaging diagnosis re-contoured the nodules in the same cases to evaluate inter-observer agreement. Both physicians were blinded to the pathological results when evaluating the images. The radiomic features from all groups demonstrated excellent inter-observer agreement (all ICC values > 0.8).A total of 851 features for the tumor, 851 for the peritumoral region, and 1702 for the combined tumor-peritumoral region were obtained. Conventional CT characteristics and radiomic features reflect lesion information from macroscopic and microscopic levels, respectively, and do not exhibit significant linear correlation (16, 17). In this study, all extracted radiomic features underwent rigorous screening, these features were then reduced using Lasso for dimensionality reduction, and the best features were selected for inclusion in the radiomics model.

In the training set, the selected radiomics features were modeled using five machine learning (ML) algorithms: logistic regression (LR), naive Bayes model, extremely randomized trees (Extra-Trees), multi-layer perceptron (MLP), and support vector machine (SVM). Ultimately, five models for each of the tumor, peritumoral, and tumor-peritumoral groups were developed, yielding a total of 15 independent radiomics models. The performance of the 15 radiomics models was compared using the receiver operating characteristic (ROC) curve. To prevent data leakage and overfitting, this study strictly ensured that all feature selection, parameter tuning, and model construction were performed exclusively within the development set. The test set and the external validation set were not involved in any training, screening, or tuning processes and were used solely for the final one-time evaluation of the model.

### 2.5 Establishment and evaluation of the clinical-subjective-radiomics combined prediction model

In the training set, a clinical-CT feature-radiomics combined prediction model was constructed using the best radiomics model, clinical features, and CT characteristics through logistic regression. The diagnostic performance of each model was evaluated in the test set and an independent external validation set. Statistical differences in The De-Long test was employed to compare differences in the area under the curve (AUC) between models. Calibration was assessed using the Hosmer-Lemeshow test and calibration curves, while the Brier score, calibration slope, and calibration intercept were calculated to quantify calibration deviation. The operational threshold was selected based on the Youden index (balancing sensitivity and specificity), and an alternative threshold with a fixed sensitivity of 95% was also compared in consideration of clinical requirements. In the training set, test set, and validation set, goodness-of-fit (Hosmer-Lemeshow) curves and clinical decision curves (DCA) were plotted to assess the model’s fit and its clinical applicability.

### 2.6 Statistical methods

Statistical analysis and model construction were performed using IBM SPSS Statistics software (version 25.0), R software (version 4.1.1), and Python (version 3.7.6). For normally distributed continuous data, independent samples t-test was used to compare differences between two groups, and the results were expressed as mean ± standard deviation. For non-normally distributed continuous data, the Mann-Whitney U test was used to compare differences between two groups, and the results were presented as median (interquartile range, IQR). Categorical data were presented as frequencies and compared using the Chi-square test or Fisher’s exact test. The diagnostic performance of models and factors was evaluated using the ROC curve. An AUC value greater than 0.9 indicates high diagnostic value, while an AUC value between 0.7 and 0.9 indicates moderate diagnostic value. *P*-value <0.05 was considered statistically significant.

## 3 Results

### 3.1 Clinical-subjective feature selection

The basic clinical information of the patients is shown in **Table 1**. Consistency analysis was performed for the features of density, vacuole sign, TVN, and DTP. The ICC values for all these features were greater than 0.75. In the training set, there were no statistically significant differences in age, gender, smoking history, or family history between the BA group and the LUAD group (*P* > 0.05). Through univariate and multivariate logistic regression analysis, the following were identified as independent factors for predicting BA and LUAD: lung lobe distribution, density, vacuolar sign, TVN, DTP, long diameter, and short diameter (**Table 2**).

**Table 1:** Clinical and CT morphological features of the training, test, and validation cohorts.

Characteristics	Training cohort (n=196)	Test cohort (n=85)	Validation cohort (n=81)	*P* value
Age (year)*	52.03 ± 12.20	56.01 ± 13.37	53.85 ± 14.22	0.057
Gender				0.449
Male	64 (32.65)	26 (30.59)	25 (30.86)	
Female	132 (67.35)	59 (69.41)	56 (69.14)	
Smoking history				0.330
Yes	36 (18.37)	14 (16.47)	9 (11.11)	
No	160 (81.63)	71 (83.53)	72 (88.89)	
Family history				0.071
Yes	16 (8.16)	4 (4.71)	1 (1.23)	
No	180 (91.84)	81 (95.29)	80 (98.77)	
Location				0.052
Left upper lobe	46 (23.47)	14 (16.47)	15 (18.52)	
Left lower lobe	47 (23.98)	13 (15.29)	15 (18.52)	
Right upper lobe	51 (26.02)	25 (29.41)	21 (25.93)	
Right middle lobe	9 (4.60)	5 (5.88)	8 (9.88)	
Right lower lobe	43 (21.93)	28 (33.95)	22 (27.15)	
Density				0.678
Solid	51 (26.02)	29 (34.12)	25 (30.86)	
Ground-glass	95 (48.47)	37 (43.53)	36 (44.44)	
Sub-solid	50 (25.51)	19 (22.35)	20 (24.69)	
Bronchial sign				
Yes	7 (3.57)	7 (8.24)	4 (4.94)	
No	189 (96.43)	78 (91.76)	77 (95.06)	
Pleural retraction sign				0.042
Yes	11 (5.61)	11 (12.94)	11 (13.58)	
No	185 (94.39)	74 (87.06)	70 (86.42)	
Vascular sign				0.153
Yes	27 (13.78)	18 (21.18)	9 (11.11)	
No	169 (86.22)	67 (78.82)	72 (88.89)	
Vacuolar sign				0.094
Yes	52 (26.53)	21 (24.71)	31 (38.27)	
No	144 (73.47)	64 (75.29)	50 (61.73)	
Cavity sign				0.416
Yes	3 (1.53)	2 (2.35)	0	
No	193 (98.47)	83 (97.65)	81 (100.00)	
Spiculated sign				0.159
Yes	11 (5.61)	7 (8.24)	10 (12.35)	
No	185 (94.39)	78 (91.76)	71 (87.65)	
Lobulated sign				0.355
Yes	2 (1.02)	2 (2.35)	2 (2.47)	
No	194 (98.98)	83 (97.65)	79 (97.53)	
DTP (mm)*	12.45 ± 12.56	9.69 ± 9.96	12.71 ± 13.17	0.169
TVN	2.53 ± 1.51	2.69 ± 1.45	2.83 ± 1.77	0.328
Long diameters (mm)*	10.45 ± 5.65	10.73 ± 6.44	10.83 ± 6.10	0.871
Short diameters (mm)*	7.85 ± 4.08	8.22 ± 4.62	8.43 ± 4.58	0.551

Except where indicated, data are numbers of participants, with percentages in parentheses. *Data are means ± SDs. DTP, distance to pleura; TVN, tumor-related vessels number.

**Table 2:** Univariate and multivariate logistic regression analysis of clinical data and conventional CT features in the training cohort.

Characteristics	Univariate logistic regression			Multivariate logistic regression		
	OR	OR (95%CI)	*P* value	OR	OR (95%CI)	*P* value
Age	0.997	0.992-1.001	0.214			
Gender	0.975	0.867-1.096	0.721			
Smoking history	0.953	0.825-1.099	0.575			
Family history	0.943	0.770-1.155	0.634			
Location	0.939	0.905-0.975	0.007	0.955	0.922-0.989	0.032
Density	1.315	1.226-1.411	0.000	1.230	1.15-1.315	<0.001
Bronchial sign	1.396	1.038-1.876	0.064			
Pleural retraction sign	0.868	0.683-1.104	0.333			
Vascular sign	1.075	0.916-1.262	0.459			
Vacuolar sign	0.848	0.749-0.960	0.029	0.860	0.77-0.96	0.024
Cavity sign	0.704	0.449-1.104	0.199			
Spiculated sign	0.956	0.752-1.217	0.759			
Lobulated sign	1.384	0.799-2.399	0.330			
DTP	1.007	1.002-1.011	0.010	1.006	1.002-1.01	0.014
TVN	1.103	1.065-1.142	0.000	1.108	1.058-1.616	<0.001
Long diameters	1.012	1.002-1.021	0.049	0.965	0.944-0.987	0.011
Short diameters	1.025	1.011-1.039	0.002	1.04	1.008-1.073	0.038

### 3.2 Establishment of the radiomics model

**Figure 4:**
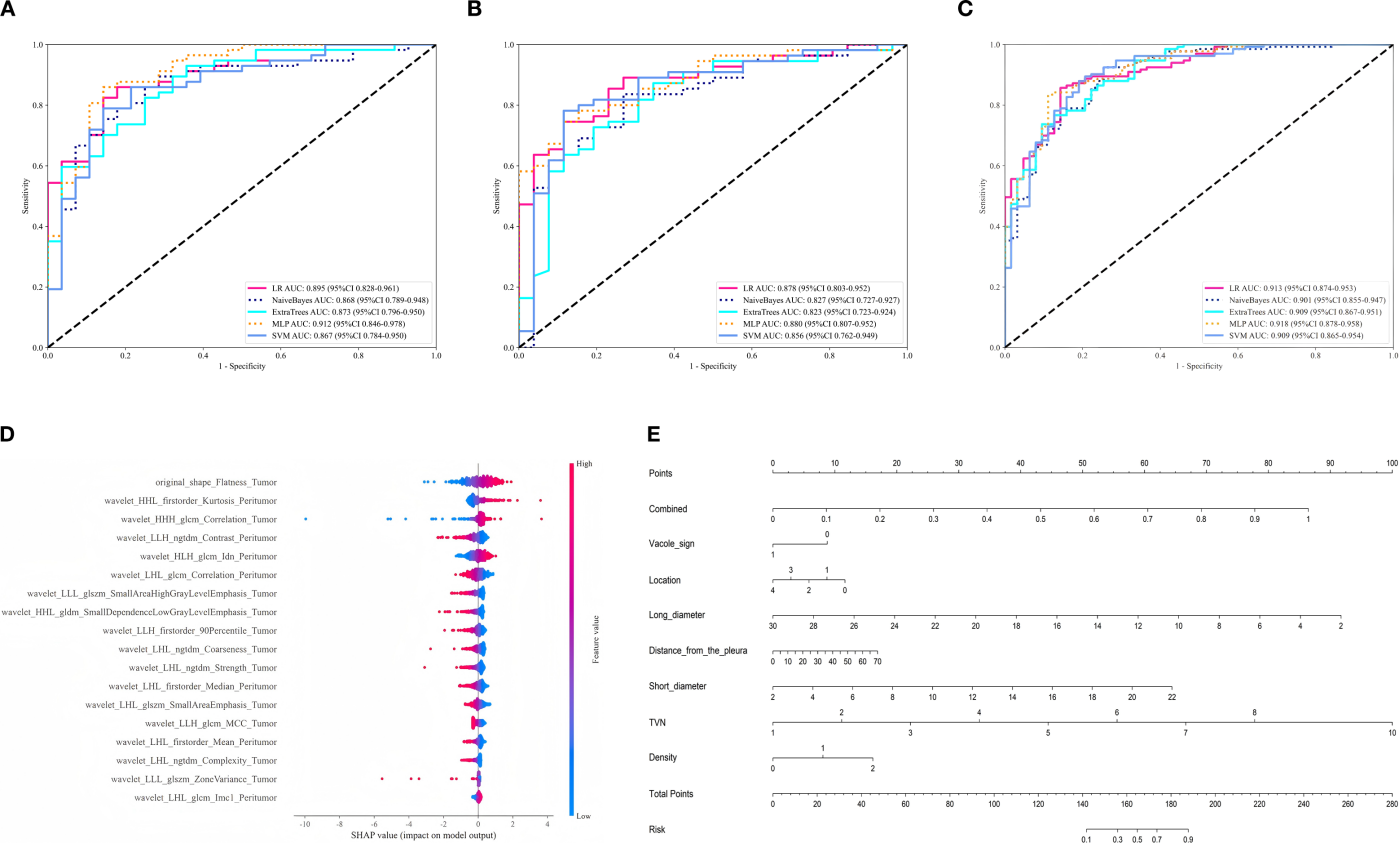
**(A-C)** ROC curves of five machine learning models for the tumor-peritumoral group in the training set, test set, and validation set. **(D)** presents the SHAP Beeswarm plot, the feature `original_shape_Flatness_Tumor` was identified as the most critical discriminating indicator. A higher degree of flatness was more indicative of lung adenocarcinoma, whereas a more rounded shape was more suggestive of bronchiolar adenoma. **(E)** Nomogram of the final fusion model.

A total of 12, 19, and 18 features were selected by Lasso regression for the tumor group, peritumoral group, and tumor-peritumoral group, respectively, as shown in **Supplementary Figure 1**; **Supplementary Table 1**. Five machine learning (ML) algorithms were then used to construct radiomics models, among these the MLP algorithm model performed the best (**Supplementary Tables 2**-**4**). The tumor-MLP model and peritumoral-MLP model was shown in **Supplementary Figures 2**, **3**. Then, the performance of the MLP model based on the tumor, peritumoral, and tumor-peritumoral were compared (**Supplementary Figures 4**, **5**). The tumor-peritumoral MLP model shown the highest AUC values: 0.918 in the training set, 0.912 in the test set, and 0.888 in the validation set, as shown in **Figure 4**; **Supplementary Table 4**. The combined-MLP model was shown in **Supplementary Figure 6**. The Shap plot of best Tumor-Peritumoral combined radiomics model with MLP shown in **Figure 4D**.

### 3.3 Construction and evaluation of the clinical-radiomics fusion model

**Figure 5:**
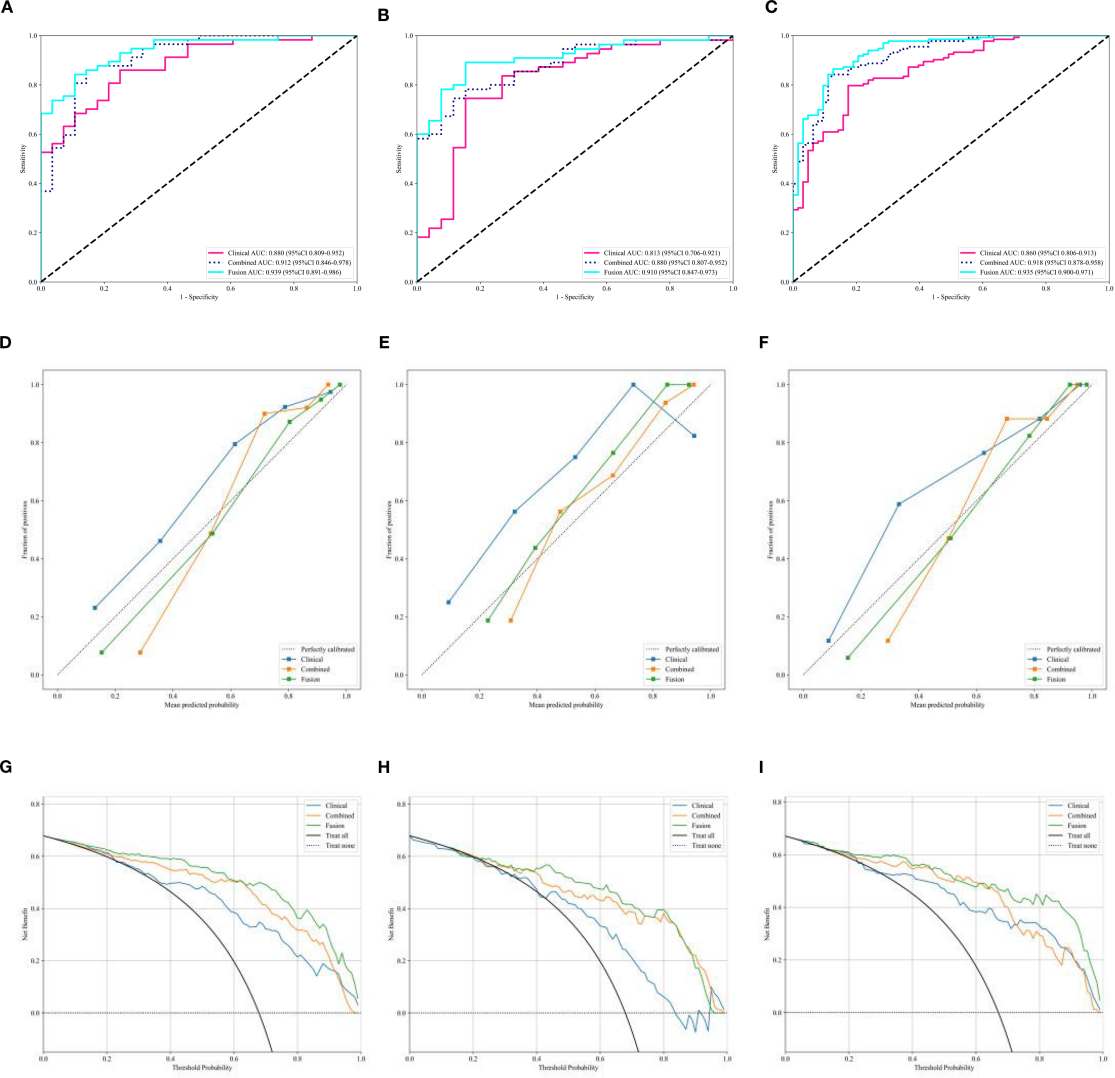
**(A-C)** ROC curves of the clinical model, tumor-peritumoral combined radiomics model, and clinical-radiomics fusion model in the training, test, and validation sets. **(D-F)** Goodness-of-fit curves of the three models in the training, test, and validation sets. **(G-I)** DCA curves of the three models in the training, test, and validation sets.

By performing logistic regression analysis, a clinical model was combined with the tumor-peritumoral radiomics combined model to construct the clinical-radiomics fusion model. The nomogram for the fusion model is shown in **Figure 4E**. The AUC values of the clinical model, the radiomics combined model, and the clinical-radiomics fusion model were compared in the training, test, and validation sets. The results are shown in **Figures 5A–C**. The De-Long test results showed that the AUC values of the clinical model, radiomics combined model, and clinical-radiomics fusion model were significantly different (*P* < 0.05) in the training, test, and validation cohorts. Among them, the clinical-radiomics fusion model achieved the highest accuracy, with AUCs of 0.862, 0.847, and 0.864, respectively (**Table 3**). The Hosmer-Lemeshow test results indicated that, except for the clinical model in the training and validation sets, the tumor–peritumor radiomics combined model and clinical-radiomics fusion model showed good fit (*P* > 0.05), as shown in **Figures 5D–F**. The DCA curves (**Figures 5G–I**) demonstrated that the clinical-radiomics fusion model yielded a higher overall net benefit than the single clinical model or radiomics model in most cases.

**Table 3:** Comparison of diagnostic performance between clinical model, tumor-peritumoral radiomics combined model, and clinical-radiomics fusion model.

Group	Model	AUC (95%CI)	Acc	Sen	Spe	PPV	NPV	De-long test P-value	
								Combined model	Fusion model
Training cohort	Clinical model	0.860 (0.806-0.913)	0.801	0.789	0.825	0.905	0.650	<0.001	0.028
	Combined model	0.918 (0.879-0.958)	0.847	0.827	0.889	0.940	0.709	/	0.048
	Fusion model	0.935 (0.900-0.971)	0.862	0.857	0.873	0.934	0.743	/	/
Test cohort	Clinical model	0.880 (0.809-0.952)	0.812	0.842	0.750	0.873	0.700	0.001	0.041
	Combined model	0.912 (0.846-0.978)	0.847	0.842	0.857	0.923	0.727	/	0.042
	Fusion model	0.939 (0.891-0.986)	0.847	0.825	0.893	0.940	0.714	/	/
Validation cohort	Clinical model	0.813 (0.706-0.921)	0.765	0.727	0.846	0.909	0.595	0.002	0.016
	Combined model	0.880 (0.807-0.952)	0.778	0.727	0.885	0.930	0.605	/	0.047
	Fusion model	0.910 (0.847-0.973)	0.864	0.873	0.846	0.923	0.759	/	/

Acc, Accuracy; Sen, Sensitivity; Spe, Specificity; PPV, Positive Predictive Value; NPV, Negative Predictive Value; Combined model, Tumor-Peritumoral Combined Radiomics Model; Fusion model, Clinical-Radiomics Fusion Model.

## 3 Discussion

BA and LUAD have significant differences in clinical treatment strategies and prognosis. However, distinguishing between the two on CT imaging and during intraoperative frozen sections remains challenging (18), making preoperative differentiation particularly difficult. In this context, this study integrated cases from two centers to systematically analyze the clinical data, CT morphological features, and tumor-peritumoral radiomics combined features of BA and LUAD. A clinical-radiomics fusion model for differential diagnosis was then constructed.

In this study, we utilized logistic regression to analyze patients’ basic clinical data and conventional CT features to screen for independent predictors for establishing a clinical-subjective model (abbreviated as the “clinical model” in the figures). However, the independent factors ultimately included in our model consisted solely of conventional CT features. While basic clinical data were insufficient for differentiating between the two entities, certain characteristics were still observed, BA predominantly occurred in middle-aged to elderly women, which is consistent with previous studies (19, 20). The average age of BA patients in this cohort was slightly older than that of LUAD patients. A similar finding was reported by Cao et al. (21), where the age of BA patients was slightly higher than that of LUAD patients. Among the CT characteristics, significant differences were observed between BA and LUAD regarding the lung lobe distribution of the nodule, density, vacuolar sign, DTP, TVN, and both the long and short diameters (*P* < 0.05). Based on these features, a clinical model was constructed. Most BA lesions are located in the lower lobes of both lungs and have a shorter DTP compared to LUAD. Previous studies have also shown that BA lesions are often located within 20 mm of the pleura (22, 23). However, a study by Sun et al. (10) found that there was little difference in the distance to the pleura between BA and LUAD, possibly due to a smaller sample size or lack of standardized measurement methods for DTP. In this study, 58.73% of BA nodules exhibited solid density, which is consistent with findings by Onishi et al. (24), who reported that BA typically presents with solid density, likely due to the presence of mucus cells in BA (25, 26). On the other hand, 58.65% of LUAD lesions showed ground-glass density, which is related to their growth pattern along the alveolar walls (27). Additionally, in this study, the size of BA lesions was generally smaller than LUAD, which is consistent with previous studies distinguishing between BA and LUAD (9, 13). The TVN of BA is also lower than that of LUAD, which may be due to the increased blood supply and associated vessel count as the tumor grade progresses (28). The vacuolar sign is more commonly observed in BA, with a study by Wang et al. (21) reporting that 50% of BA cases presented with this sign. This could be because BA grows and pulls on surrounding tissues, leading to the dilation of adjacent bronchi (22, 29), or because partial bronchial obstruction around BA causes the expansion of nearby alveolar spaces (30).

In this study, not only the tumor region was delineated, but the peritumoral region was also automatically expanded by 3 mm. Based on the weight analysis of peritumoral radiomic features in LUAD and BA, this study identified two categories of core discriminatory features: positive-weight features, represented by `original_shape_Elongation_Peritumor` and `wavelet_LHL_glcm_Imc1_Peritumor`, which were significantly overexpressed in LUAD and correspond to peritumoral microscopic invasion, stromal remodeling, and inflammatory response, the pathological diagram of LUAD is shown in **Supplementary Figure 7**; and negative-weight features, represented by `wavelet_LLL_ngtdm_Coarseness_Peritumor`, which were significantly overexpressed in BA and reflect the structural integrity of the surrounding normal lung tissue, the pathological diagram of BA is shown in **Supplementary Figure 7**. These features quantify the pathological differences in the peritumoral microenvironment between the two lesion types, providing an objective imaging basis for preoperative non-invasive differential diagnosis and assessment of LUAD aggressiveness. Jaileene et al (14) constructed a prognostic model for lung cancer based on the radiomic features of both the tumor and the expanded 3 mm peritumoral regions, which significantly improved prediction accuracy compared to a model using only the tumor region. Similarly, in the radiomics studies on esophageal cancer and liver cancer, researchers have used both tumor and peritumoral features for modeling (31, 32). Models integrating tumor and peritumoral radiomics features generally show better diagnostic performance. In this study, the tumor–peritumor radiomics combined model also performed better overall, with the model constructed based on the MLP algorithm yielding the best diagnostic efficacy. Therefore, this model was ultimately selected for the construction of the clinical-radiomics fusion model for subsequent clinical applications.

This study integrated clinical CT features, including lung lobe distribution, density, vacuolar sign, TVN, DTP, and nodule long and short diameters, with the optimal radiomics model. A combined model was constructed through logistic regression analysis and a nomogram was created to predict LUAD. The ROC curve indicated that the combined model had the highest diagnostic efficacy. Previous studies, such as the one by Sun et al. (10), showed that radiomics models for diagnosing microinvasive lung adenocarcinoma outperformed conventional CT features, with AUC values of 0.976 and 0.708, respectively. Similarly, Liu et al. (13) demonstrated that a nomogram integrating CT image features and radiomics features for differentiating BA from LUAD showed highest, with AUC values of 0.901, 0.854, and 0.769, respectively. Models constructed using both tumor and peritumoral radiomics features generally demonstrated higher efficacy. One study on predicting LUAD dissemination along the airways using radiomics features reported that the tumor–peritumor combined model had the best efficacy, with an AUC of 0.985, specificity of 0.937, and sensitivity of 0.938 (33), which aligns with the findings of this study. The DCA curve showed that within the low threshold range (0–0.6), the net benefit of the fusion model and the optimal radiomics model was slightly higher than that of the clinical-subjective model, with the fusion model performing best; In the medium-to-high threshold range (0.6–0.85), the net benefit advantage of the fusion model further increased, becoming significantly higher than that of the other two models, This indicates that the fusion model can maintain the highest net benefit even under more conservative or more precise treatment decision-making scenarios, likely because the combination of CT features and tumor–peritumor radiomics features offers a deeper insight into tumor heterogeneity and peritumoral microenvironment changes. Based on the above, the clinical-radiomics fusion model is the best model for differentiating BA from LUAD in this study.

## 4 Conclusion

In this study, lung lobe distribution, density, vacuolar sign, tumor-related vessel number, DTP, nodule long and short diameters showed certain value in differentiating BA from LUAD. Among the CT radiomics models constructed using various ML algorithms, the tumor–peritumor radiomics combined model demonstrated higher predictive efficacy than the models based on tumor-only or peritumor-only features. The clinica-radiomics fusion model provided the best performance in distinguishing BA from LUAD, and it has the potential to serve as a non-invasive and efficient preoperative tool for differentiating these two conditions, offering important reference for clinical decision-making.

However, this study has some limitations. This study included cases from two centers with variations in scanning equipment and parameters, which may introduce some bias into the results. Due to its retrospective nature, there is inherent selection bias. Additionally, LUAD patients were not classified according to specific pathological types including *in situ* adenocarcinoma, microinvasive adenocarcinoma, invasive adenocarcinoma. Currently, our model has certain limitations in distinguishing morphologically similar BA from MIA. Future research may involve more in-depth studies, potentially integrating biomarkers to enhance model performance, will further differentiate various types of LUAD from BA.

## Data Availability

The raw data supporting the conclusions of this article will be made available by the authors, without undue reservation.
