# Supplementary material for: A fusion model based on tumor and peritumoral CT radiomics for differentiating bronchiolar adenoma from lung adenocarcinoma
Source: Front Oncol. 2026 Apr 15;16:1798218. doi: 10.3389/fonc.2026.1798218 (PMC13124503; doi:10.3389/fonc.2026.1798218)
Supplement: Supplementary file 1 [file Supplementaryfile1.docx]

**Supplementary Materials**

| 1A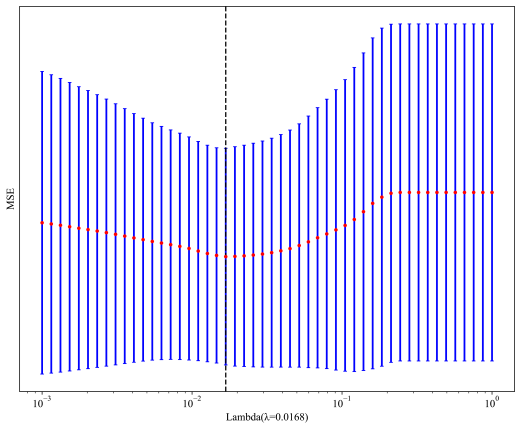 | 1B 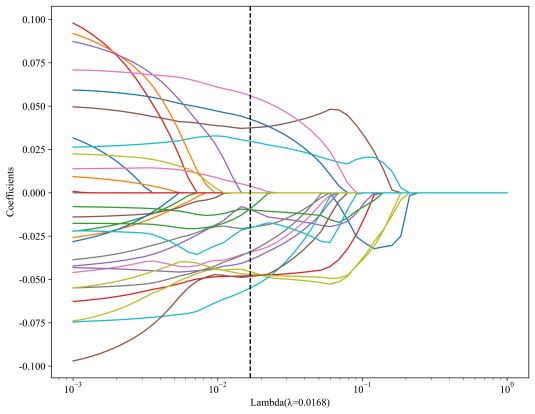 |
| --- | --- |
| 1C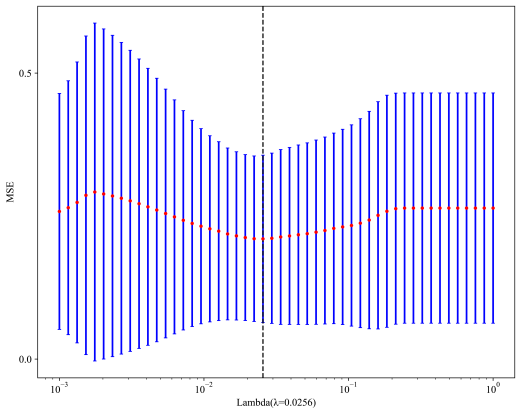 | 1D 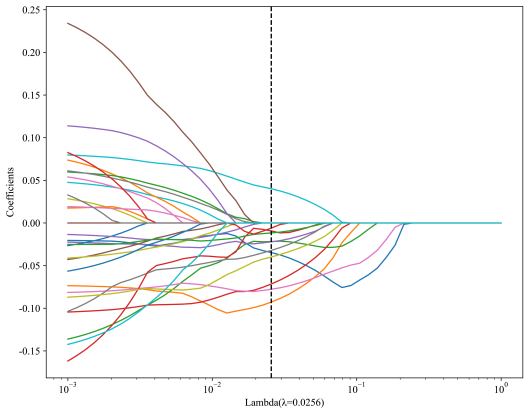 |
| 1E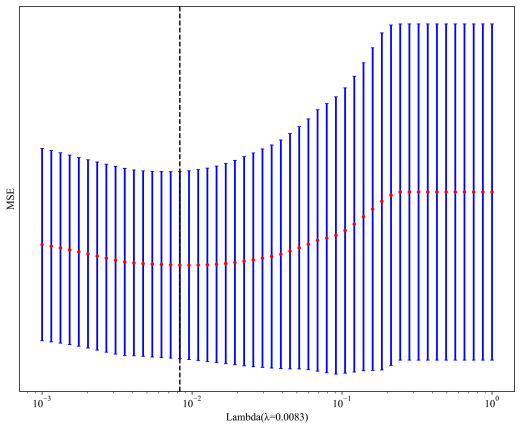 | 1F 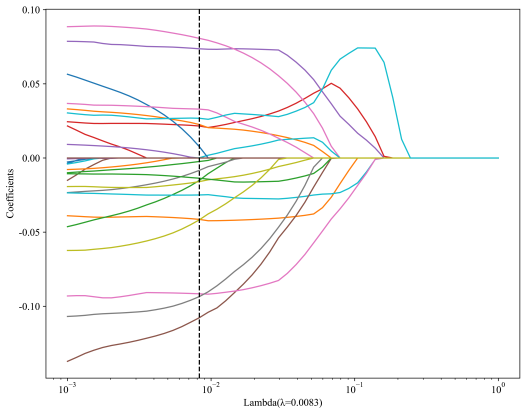 |

Supplementary Figure 1: Process of feature selection using LASSO regression. (A) and (B), (C) and (D), (E) and (F) represent the process of selecting the optimal radiomics features for the tumor-peritumoral group, tumor group, and peritumoral group, respectively. (A), (C), and (E) show the optimal Log(λ) values obtained through 10-fold cross-validation to minimize model bias, with the vertical dashed line representing the best Lambda value. (B), (D), and (F) illustrate the relationship between the coefficients of different radiomics features and λ, with the dashed line indicating the Lambda value selected based on 10-fold cross-validation.


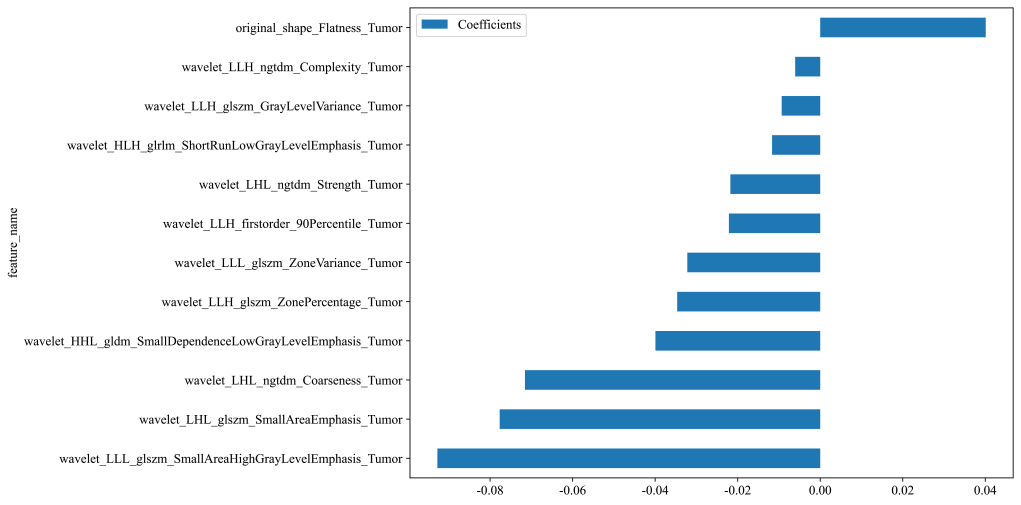


Supplementary Figure 2: Radiomics features and their weight coefficients used for modeling in the tumor CT radiomics characteristics.


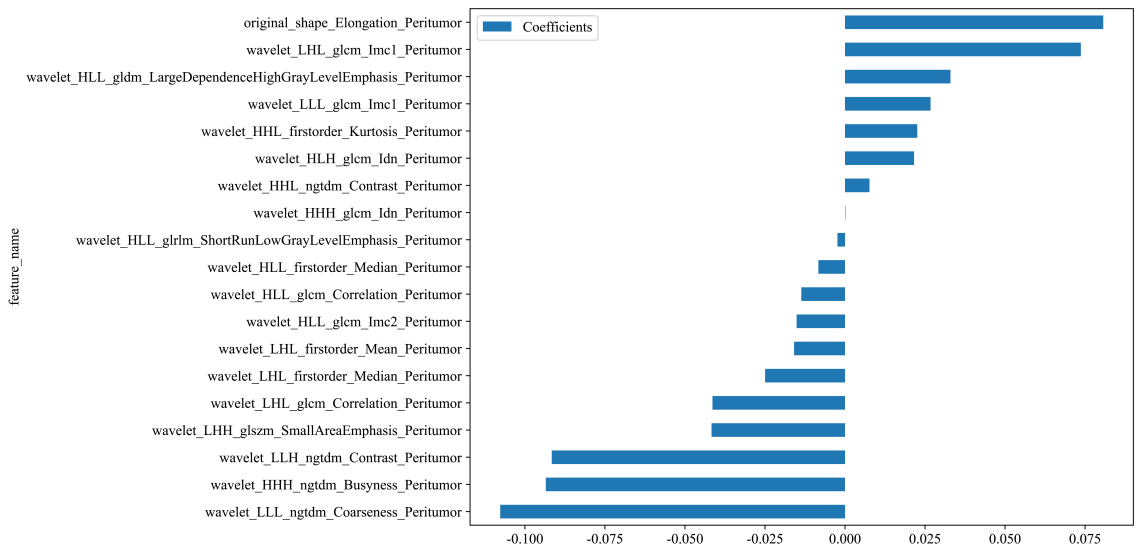


Supplementary Figure 3: Radiomics features and their weight coefficients used for modeling in peritumoral CT radiomics characteristics.

| A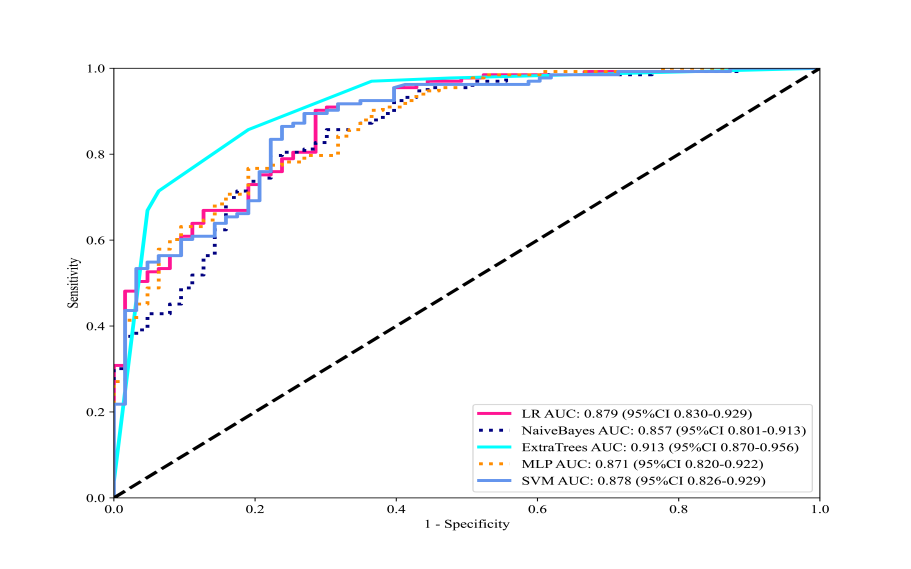 |
| --- |
| B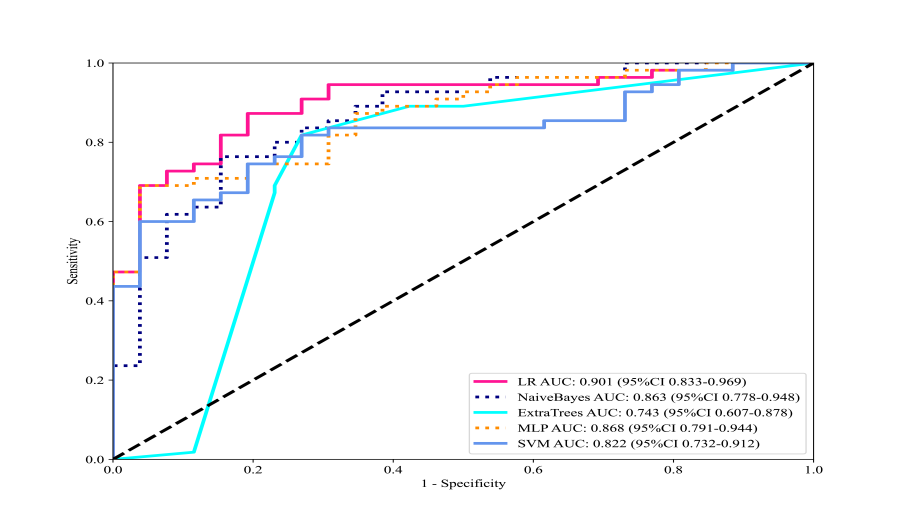 |
| C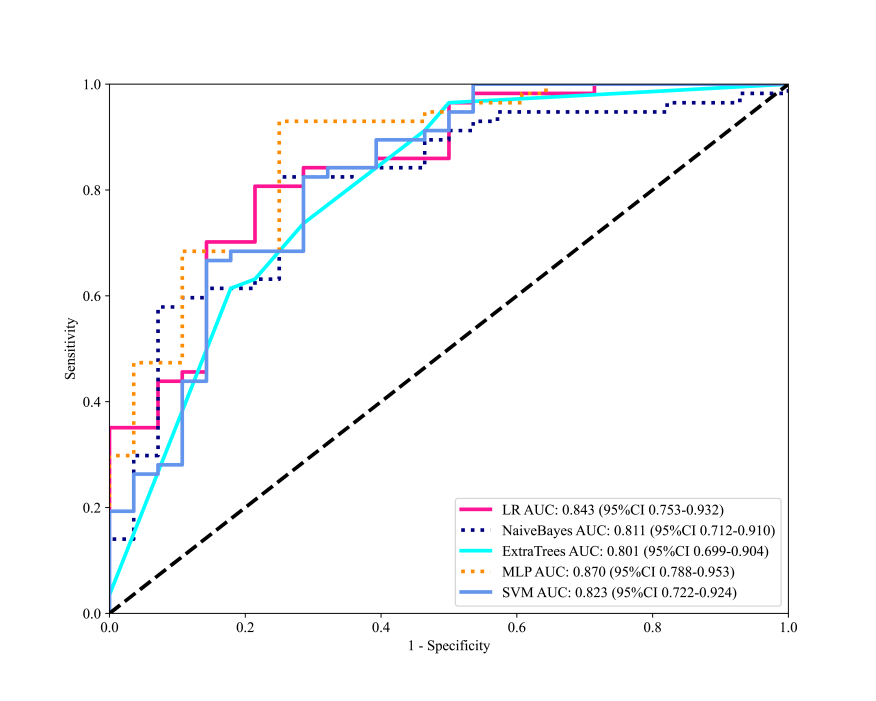 |

Supplementary Figure 4: ROC curves of different machine algorithm radiomics models for the tumor group in the training, test, and validation sets (A-C).

| A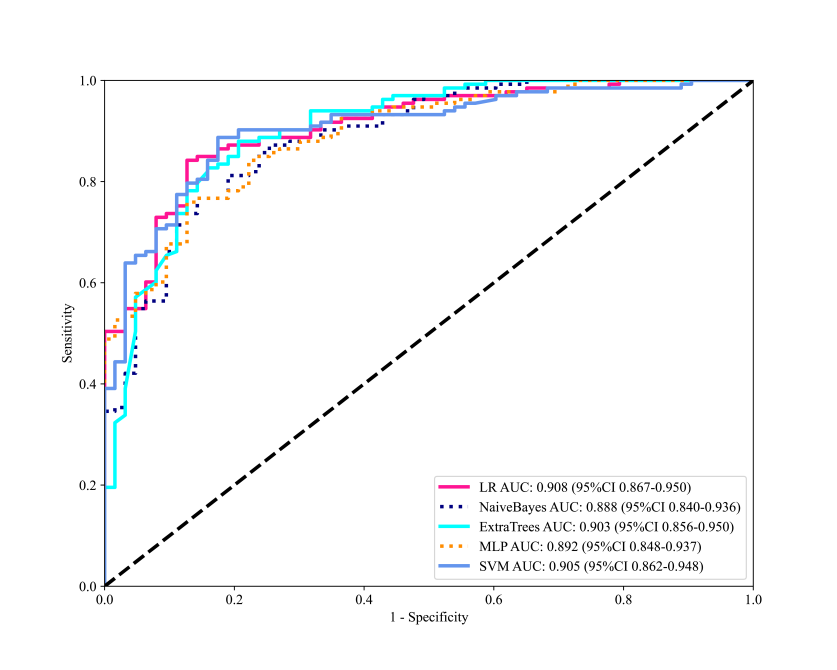 |
| --- |
| B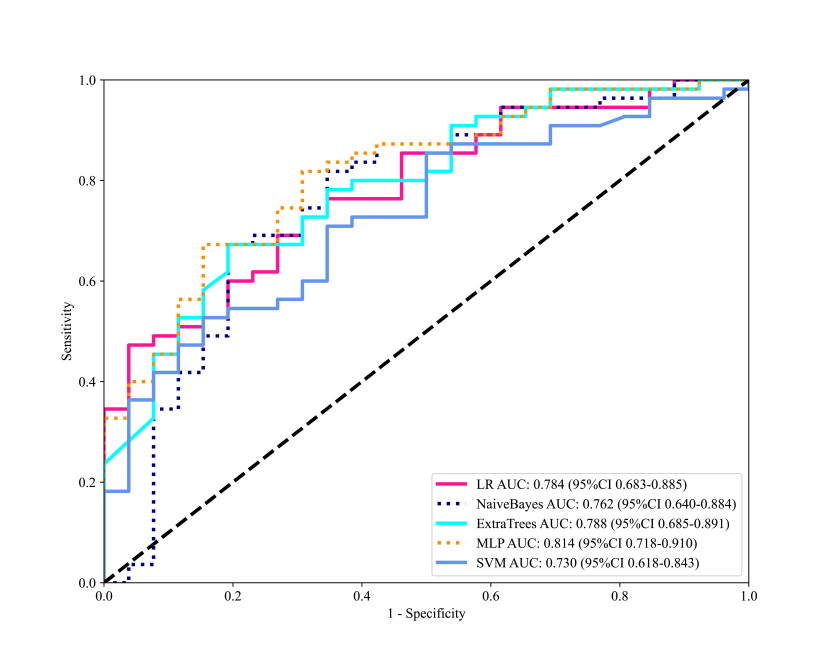 |
| C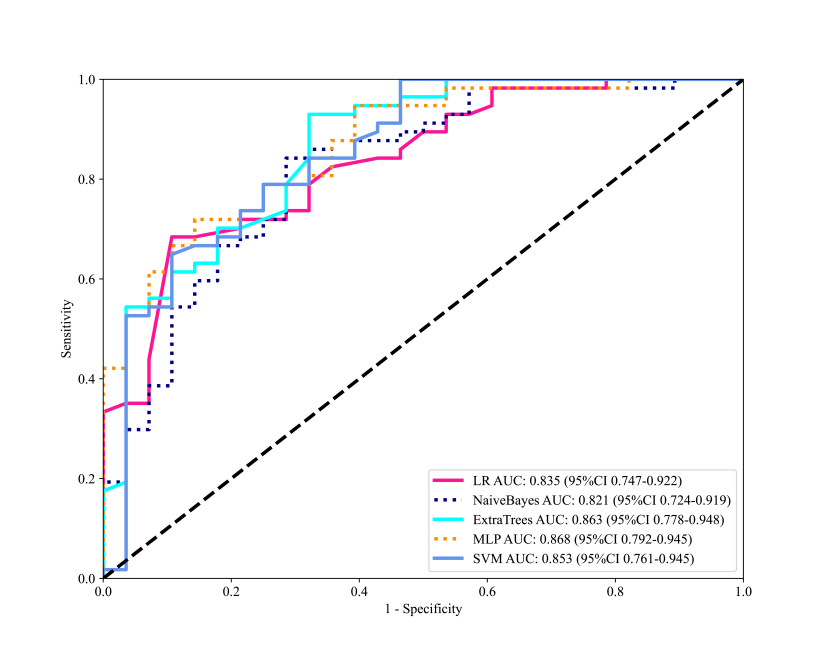 |

Supplementary Figure 5: ROC curves of radiomics models for different machine algorithms in the peritumoral group in the training set, test set, and validation set (A-C).

| 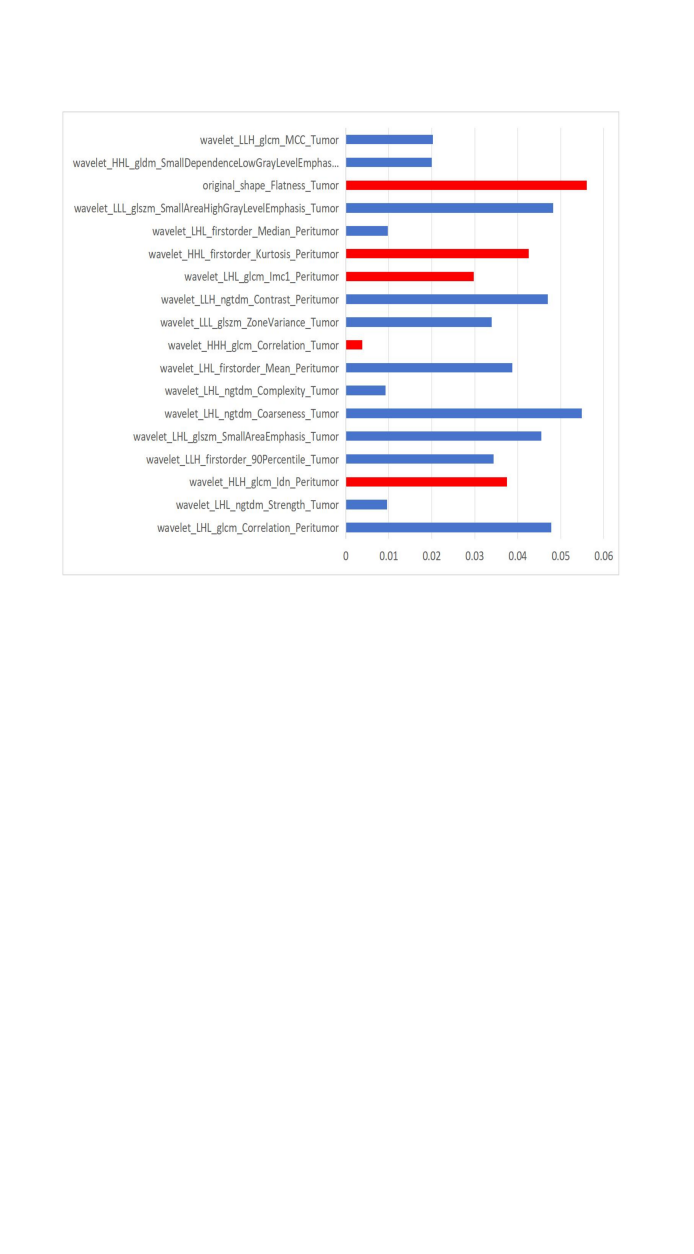 |
| --- |
| B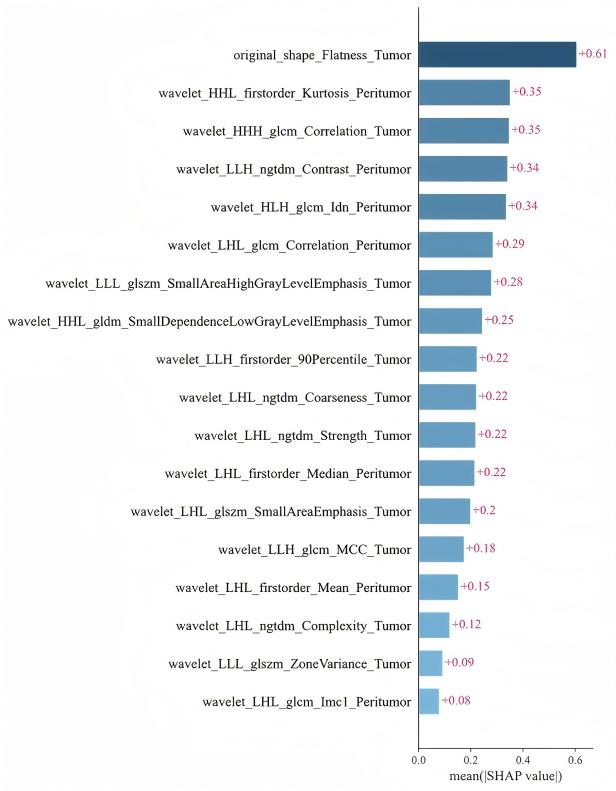 |

Supplementary Figure 6: A. The feature coefficients included in the best radiomics model scoring formula, with blue representing negative values and red representing positive values. B presents the SHAP Bar plot of the best radiomics model.

| A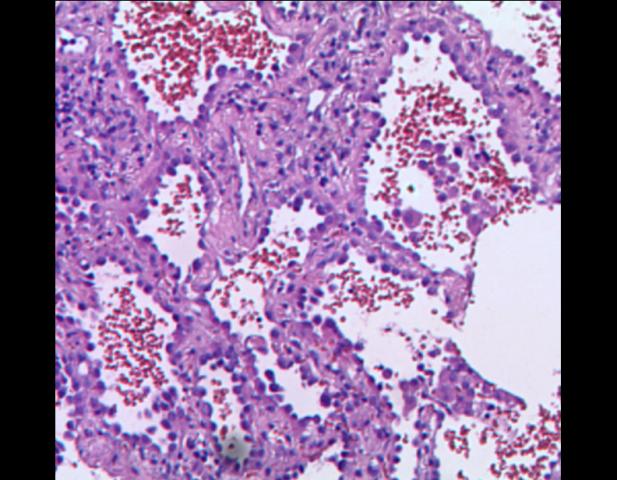 | B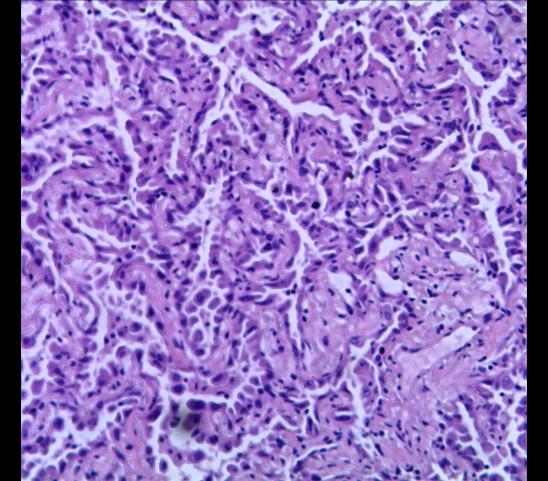 |
| --- | --- |

Supplementary Figure 7: A shows a pathological image of bronchiolar adenoma (hematoxylin and eosin [H&E] stain, 200× magnification), demonstrating bland cytology, no significant atypia, a continuous basal cell layer, and absence of invasive growth. B shows a pathological image of lung adenocarcinoma (H&E stain, 200× magnification), revealing invasive atypical glands, marked cytological atypia, and a desmoplastic stromal reaction.

| A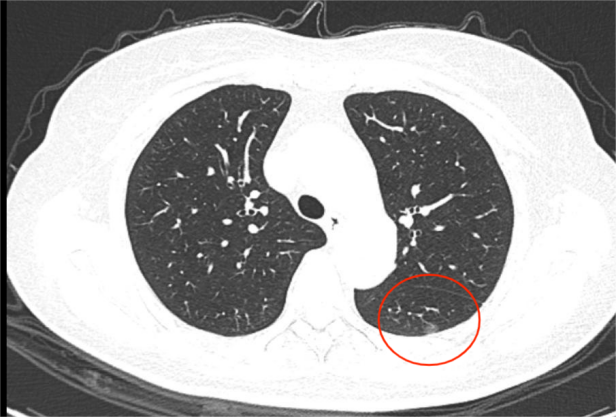 | B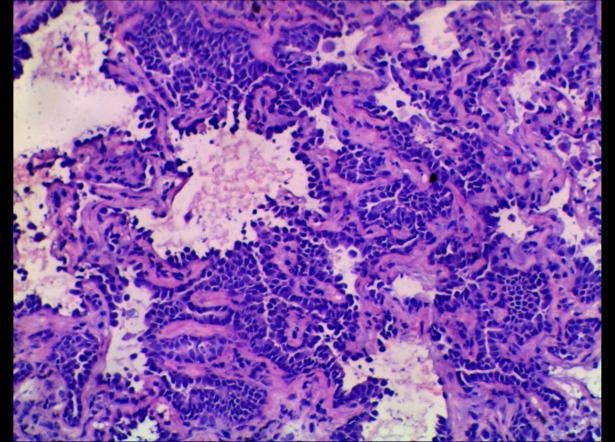 |
| --- | --- |

Supplementary Figure 8: A shows a non-contrast chest CT lung window image of a 53-year-old female patient. The tumor-to-vessel number (TVN) was 3, the distance to the pleura (DTP) was 0 mm, the radiomics score was approximately 0.56, with a long-axis diameter of 7 mm and a short-axis diameter of 6 mm. When the relevant features were input into the fused model nomogram, the predicted probability of lung adenocarcinoma (LUAD) was low. However, postoperative pathological examination (B) revealed that the pulmonary nodule in the dorsal segment of the left lower lobe was a minimally invasive adenocarcinoma (MIA), indicating a misclassification by the model.

The fused model demonstrated good performance in differentiating between lung adenocarcinoma and bronchiolar adenoma (BA). However, diagnostic challenges persist in some cases, primarily due to the significant overlap in their imaging phenotypes. This is particularly true for lepidic-predominant, well-differentiated lung adenocarcinomas, which can perfectly mimic the benign appearance of BA on imaging. Both entities often present as nodules ≤2 cm, with smooth margins, minimal pleural indentation, and relatively homogeneous density, lacking typical malignant signs such as spiculation, cavitation, or heterogeneous enhancement. Consequently, macroscopic imaging features are often insufficient for reliable distinction.

Secondly, the small lesion volume limits the stability of radiomic features. The region of interest (ROI) is susceptible to noise and partial volume effects, reducing the reproducibility of texture and intensity-based features. Furthermore, radiomic features cannot capture the microscopic pathological differences that determine malignant potential, such as cellular atypia, basilar membrane invasion, or loss of cellular polarity. Both well-differentiated lung adenocarcinoma and BA exhibit low tumor heterogeneity. The subtle architectural differences distinguishing benign hyperplasia from early invasive carcinoma are often below the resolution threshold of CT imaging, further diminishing the discriminative power of radiomic features.

Additionally, the scarcity of borderline cases in the training dataset may constrain the model's generalizability. Machine learning algorithms struggle to learn the discriminative patterns for lesions in this ambiguous zone. Consequently, uncertainty remains in the radiomics-based differentiation of some lung adenocarcinoma-BA cases. This highlights the need to incorporate biomarkers or advanced imaging techniques to optimize the stratified diagnosis of these challenging cases.
